# Endogenously Expressed IL-4Rα Promotes the Malignant Phenotype of Human Pancreatic Cancer In Vitro and In Vivo

**DOI:** 10.3390/ijms18040716

**Published:** 2017-03-28

**Authors:** Benno Traub, Lie Sun, Yongsu Ma, Pengfei Xu, Johannes Lemke, Stephan Paschke, Doris Henne-Bruns, Uwe Knippschild, Marko Kornmann

**Affiliations:** 1 Department of General and Visceral Surgery, University of Ulm, Albert-Einstein-Allee 23, 89081 Ulm, Germany; benno.traub@uniklinik-ulm.de (B.T.); sunliemd@126.com (L.S.); mys870311@sina.com (Y.M.); pegfei.xu@uniklinik-ulm.de (P.X.); johannes.lemke@uniklinik-ulm.de (J.L.); stephan.paschke@uniklinik-ulm.de (S.P.); doris.henne-bruns@uniklinik-ulm.de (D.H.-B.); uwe.knippschild@uniklinik-ulm.de (U.K.); 2 Department of General Surgery, Peking University First Hospital, 8th Xishiku Street, Xicheng, Beijing 100034, China

**Keywords:** interleukin-4-receptor, IL-4-receptor-α-chain, IL-13-receptor-α1-chain, interleukin-4, pancreatic cancer

## Abstract

Exogenous interleukin-4 (IL-4) has been demonstrated to affect the growth of different human malignancies including pancreatic cancer cells. The aim of our study was to determine the role of endogenously expressed IL-4-receptor-α-chain (IL-4Rα) in pancreatic cancer cells. IL-4Rα-suppression was achieved by generating Capan-1 cells stably expressing shRNA targeting IL-4Rα. The malignant phenotype was characterized by assessing growth properties, directional and non-directional cell movement in vitro and tumor growth in vivo. Signaling pathways were analyzed upon IL-4 and IL-13 stimulation of wildtype (WT) and control-transfected cells compared to IL-4Rα-knockdown cells. Silencing of IL-4Rα resulted in reduced anchorage-dependent cell growth (*p* < 0.05) and reduced anchorage-independent colony size (*p* < 0.001) in vitro. Moreover, cell movement and migration was inhibited. IL-4 and IL-13 stimulation of Capan-1-WT cells induced activation of similar pathways like stimulation with Insulin-like growth factor (IGF)-I. This activation was reduced after IL-4Rα downregulation while IGF-I signaling seemed to be enhanced in knockdown-clones. Importantly, IL-4Rα silencing also significantly suppressed tumor growth in vivo. The present study indicates that endogenously expressed IL-4 and IL-4Rα contribute to the malignant phenotype of pancreatic cancer cells by activating diverse pro-oncogenic signaling pathways. Addressing these pathways may contribute to the treatment of the disease.

## 1. Introduction

Human pancreatic cancer ranks fourth among all cancer-related mortalities with very limited improvement in five-year overall survival in the last three decades [1]. Multiple genetic alterations and deregulation of several growth factors and their receptors have been identified [2]. Their associated pathways got into the focus of researchers and physicians due to their widespread relevance: similar to other malignancies like gastric and colorectal cancer, extensive work was performed in order to determine the core pathways of pancreatic carcinogenesis [3]. 

The immune-modulatory cytokine interleukin-4 (IL-4) and its associated receptor chains interleukin-4-receptor-α (IL-4Rα) and interleukin-13-receptor-α1 (IL-13Rα1) are not known in the first place for their growth-promoting potential. Nevertheless, both ligand and receptor chains have been shown to be overexpressed in pancreatic cancer [4,5]. IL-4, mainly produced by CD4+ T-cells [6], binds to its transmembrane receptor chain (IL-4Rα), a 140 kDa protein. The subsequent association with either the common γ-chain (γc) or the 47 kDa IL-13Rα1-chain forms the type-I-IL-4-receptor (γc) or the type-II-IL-4-receptor (IL-13Rα1) [7]. On non-haematopoietic cells, the type-II-IL-4-receptor (IL-4/IL-4Rα/IL-13Rα1) represents the predominant IL-4 receptor [7].

Interestingly, intracellular signaling is dependent on activation of receptor-associated kinases of the Janus-kinase (JAK) family and downstream activation of the insulin-receptor-substrates (IRS) 1/2 and the signal-transducer-and-activator-of-transcription- (STAT-) family, both overexpressed and deregulated in pancreatic cancer [8]. 

Activation of IRS-1 and -2 induces signaling transmission through messenger kinases, including K-RAS (Kirsten rat sarcoma viral oncogene), the most common gain-of-function mutation in pancreatic cancer. Parallel thereto, the JAK/STAT-pathway may contribute to tumor progression, especially with activation of STAT3 [9] 

Consequently, it is only little surprising that the expression of IL-4 and IL-4Rα seems to be associated with malignant transformation in several tumor types. First, as stated above, IL-4 can exert growth-stimulatory and pro-invasive effects in several cancer cells including the pancreas [10,11,12]. Second, IL-4Rα is over-expressed in several human tumors [13]. Third, IL-4 protein is found abundantly in the surroundings of tumor cells, secreted by infiltrating lymphocytes [14] as well as by the tumor cells themselves [11,12]. However, also opposing growth inhibitory and anti-invasive effects in human malignancies have been reported [15,16], which suggests that the effects of IL-4 in human malignancies may be cell type and tissue specific. Previous studies by our group have shown the presence and biological responsiveness of the IL-4-receptor in pancreatic cancer cells by growth inhibition induced by *Pseudomonas exotoxin* coupled to IL-4, as well as growth promotion by exogenous IL-4 in pancreatic cancer cells [5,12]. 

Interestingly, all previous studies used an exogenous approach within their experiments. In contrast, the endogenous activation of IL-4Rα in pancreatic cancer, either by auto- and paracrine IL-4 stimulation or by constitutive activation of IL-4Rα and its effects has not yet been examined. Thus, the aim of the present study was to determine the role of IL-4Rα expression in the progression of human pancreatic cancer cells.

## 2. Results

### 2.1. Expression of Type-II IL-4R Chains in Pancreatic Cancer Cells

**Figure 1:**
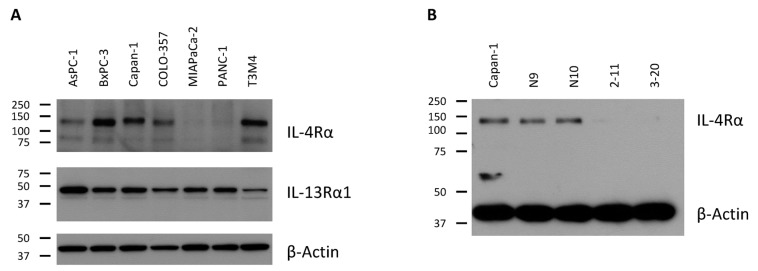
Expression of the type-II-IL-4-receptor in human pancreatic cancer cells. (**A**) interleukin-4-receptor-α (IL-4Rα) (140 kDa) and interleukin-13-receptor-α1 (IL-13Rα1) (47 kDa) protein in pancreatic cancer cell lines; (**B**) Transfection of Capan-1 with an IL-4Rα-shRNA construct strongly inhibited protein expression in the target clones 2-11 and 3-20.

All tested pancreatic cancer cell lines AsPC-1, BxPC-3, Capan-1, COLO-357, MIAPaCa-2, PANC-1 and T3M4 expressed both IL-4Rα (140 kDa) and IL-13Rα1 (47 kDa) at various expression levels (Figure 1A). The second band for IL-4Rα at 90 kDa was shown to be the non-glycosylated IL-4Rα by incubation of Capan-1-cells with Tunicamycin, an inhibitor of N-linked glycosylation (Figure S1A).

In order to test the in vitro and in vivo influence of IL-4Rα on pancreatic cancer cells, we planned to establish clones with IL-4Rα-downregulation on protein level. Although Capan-1 did not show highest IL-4Rα expression, it was chosen for down-regulation of IL-4Rα due to its good mitogenic response to exogenous IL-4 [12]. Screening of individual clones revealed highest efficacy of IL-4Rα downregulation in clones 2-11 and 3-20. Sham-transfected clones N9 and N10 showed no difference in IL-4Rα expression and were used as control clones in further experiments (Figure 1B). Screening of IL-13Rα1-expression in IL-4Rα knockdown cells showed no difference in IL-13Rα1 expression (Figure S1B). RT-PCR showed no difference in RNA-expression after transfection, indicating an inhibition of translation but not transcription (Figure S1C).

### 2.2. Effect of IL-4Rα Inhibition on Basal In Vitro Cell Growth

**Figure 2:**
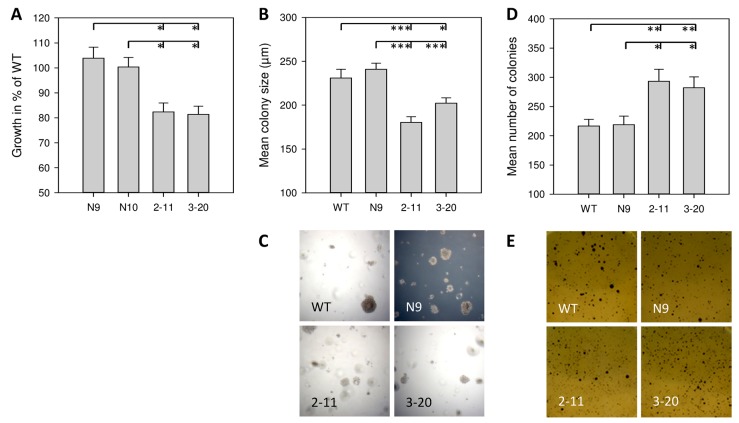
Basal growth of Capan-1 wild-type (WT), sham transfected and IL-4Rα-downregulated cells. (**A**) Basal anchorage dependent growth in the MTT assay. Data are shown as mean growth in % (±SEM) compared to WT and are means of 3 independent experiments of quadruplicate determinations; (**B**–**E**) Anchorage-independent growth. Cells were grown in soft agar and evaluated after 21 days; (**B**) Mean colony diameter in µm (±SEM) measured at 6 random positions per well with (**C**) representative examples using 4× magnification; (**D**) Vital colonies in 4 cm^2^ and (**E**) representative examples (1.2× magnification). The soft agar assay was performed as 4 independent experiments of triplicate determinations * *p* < 0.05, ** *p* < 0.01, *** *p* < 0.001.

Capan-1 cells have been shown to secrete endogenous IL-4 [12]. Inhibition of IL-4Rα and, by that, disruption of IL-4/IL-4Rα-signaling, resulted in reduced basal anchorage-dependent growth of about 20%. In detail, the growth of clone 2-11 (82.4% ± 3.6% SEM compared to WT cell growth) and clone 3-20 (81.4% ± 3.3% SEM compared to WT cells) was significantly reduced compared to control cells of WT, N9 and N10 (*p* < 0.05, Figure 2A). 

To determine anchorage-independent growth abilities, colony number and size were measured in the soft agar assay. Colonies of WT (231.1 ± 9.9 µm SEM) and N9 (240.9 ± 7.0 µm SEM) were larger than those of 2-11 (180.3 ± 6.6 µm SEM, *p* < 0.001) and 3-20 (202.1 ± 6.1 µm SEM, *p* = 0.014 vs. WT, *p* < 0.001 vs. N9), respectively (Figure 2B,C). Interestingly, after staining of vital colonies with MTT-solution (5 mg/well), the number of vital colonies in the screened area (4 cm^2^) was higher in 2-11 (293.2 ± 20.5 SEM) and 3-20 (282.3 ± 18.6 SEM) compared to WT (216.6 ± 11.4 SEM) and N9 (218.8 ± 14.8 SEM) (*p* < 0.05).

Cell cycle analysis after IL-4Rα-knockdown showed no difference in the cell cycle progression and no difference regarding apoptosis (Figure S2).

### 2.3. Effect of IL-4Rα Inhibition on Cancer Cell Motility

**Figure 3:**
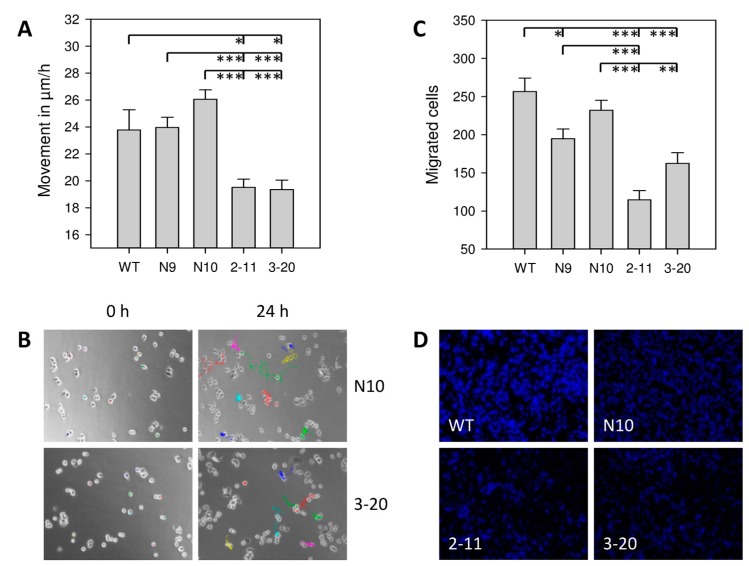
Cell movement. (**A**) Non-directional movement was tracked for 24 h using video time-lapse microscopy. Results are shown as the mean movement (in µm/h ± SEM) of 3 independent experiments evaluating each 40 cells; (**B**) Exemplary cell tracking over 24 h of single cells for N10 and 3-20 cells, 40× magnification; (**C**) Directional migration in the modified Boyden chamber. Results are shown as number of migrated cells after 36 h (±SEM) and are means of 3 separate experiments of triplicate determinations; (**D**) Representative areas of migrated cells at 10× magnification. * *p* < 0.025, ** *p* = 0.002, *** *p* < 0.001.

To assess effects of IL-4Rα on motility, non-directional movement was tracked for 24 h using video time-lapse microscopy. WT, N9, and N10 cells covered 23.8 ± 1.5 µm/h SEM, 24.0 ± 0.8 µm/h, and 26.1 ± 0.7 µm/h, respectively. IL4Rα downregulation significantly reduced the cell mobility to 19.5 ± 0.6 µm/h (2-11) and 19.4 ± 0.7 µm/h (3-20), respectively (Figure 3A,B). Likewise, IL-4Rα downregulation impaired directed migration in the Boyden chamber assay of 2-11 (−55.3%, −41.1% and −50.6% compared to WT, N9 and N10 (*p* < 0.001)) and 3-20 (−36.8% (*p* < 0.001), −16.7% (*p* = 0.105) and −30.1%, (*p* = 0.002) compared to WT, N9 and N10) (Figure 3C,D). Despite the altered migration abilities no differences in cytoskeleton arrangement were observed with Phalloidin staining of actin fibers (Figure S3).

### 2.4. Effect of IL-4Rα Downregulation on IL-4 and IL-13 Signaling

Next, the effects of IL-4Rα downregulation on signaling were investigated. Exogenous IL-4 (5 nM for 5 min) induced strong phosphorylation of several pro-oncogenic pathways in Capan-1 wildtype cells, mostly in c-Jun, ERK-1/2, and STAT3. Generally, IL-4 induced stronger protein phosphorylation compared to IL-13. The potent mitogen IGF-I was used as comparison with similar results as obtained for IL-4 and IL-13.

To determine the effects of IL-4Rα downregulation, intracellular signaling was compared in clones 3-20 and N10. Especially the phosphorylation of Akt, p38 and S6 was reduced in the IL-4Rα knockdown clone 3-20 after IL-4 stimulation. The most prominent reduction was shown in Akt-kinases. Interestingly, IGF-I-induced phosphorylation tends to be more prominent in 3-20 compared to N10. Again, this effect was strongest for Akt kinases.

**Figure 4:**
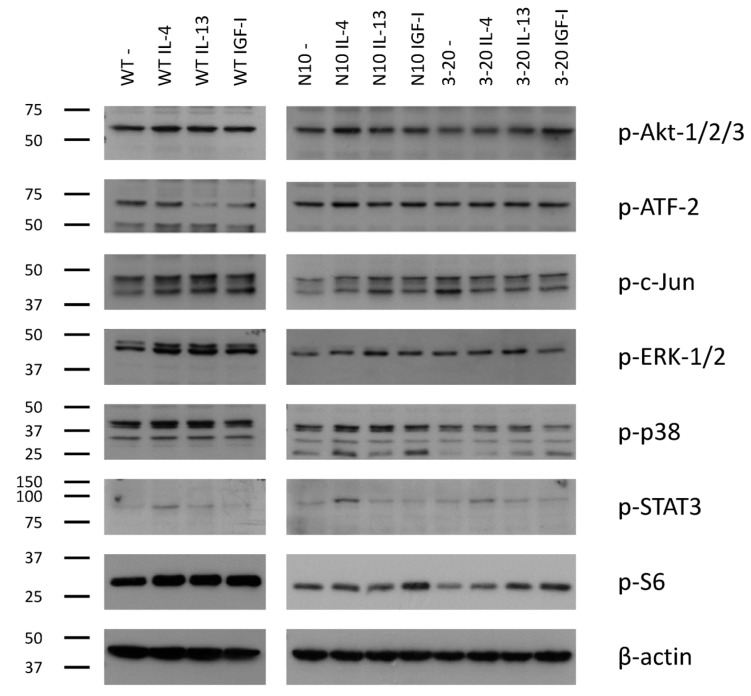
Effect of IL-4Rα downregulation on protein phosphorylation. Protein phosphorylation in cytokine (IL-4, IL-13, IGF-I) treated Capan-1 WT (left) and clones N10 and 3-20 (right) (5 nM for 5 min) in comparison to non-treated (−) control cells are shown for the indicated proteins (listed on right side).

IL-13 signaling didn’t seem to be affected by IL-4Rα downregulation (Figure 4).

The Western-Blots were analyzed quantitatively using ImageJ 1.47v. The calculation is shown in Figure S4, the results are shown in the supplementary Figure S5 and Table S1 (Stimulation in WT-cells) and Figure S6 and Table S2 (Comparison of 3-20 and N10).

### 2.5. Inhibition of STAT3 Results in Reduced Tumor Cell Survival

**Figure 5:**
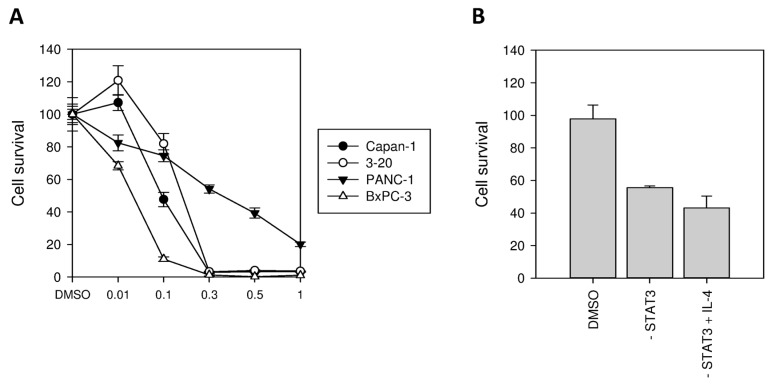
Effect of STAT3 inhibition on pancreatic cancer cell survival. Significant inhibition was achieved at 0.1 µM in different cell lines ((**A**), Note: For reasons of displayability, the shown scale in µM is not linear). Exogenous IL-4 was not able to antagonize the effects of STAT3 inhibition, *LLL12* alone (0.3 µM) and STAT3 inhibitor in combination with IL-4 (5 nM) showed no differences in cell survival of PANC-1 wildtype cells (**B**). Data are shown as means of two independent experiments of quadruplicate determinations.

Being an important messenger in IL-4 signaling, we tested the effects of STAT3 inhibition in dependency of IL-4Rα expression. Therefore we used the specific inhibitor of STAT3-phosphorylation *LLL12*. Cells of BxPC-3, Capan-1, and PANC-1 wildtype as well as IL-4Rα knockdown cells 3-20 were incubated with rising concentrations of *LLL12* from 0.01 µM up to 1 µM for 48 h. *LLL12* showed a dose-dependent inhibition of tumor cell survival in the MTT-Assay which already reached the level of significance at low doses of 0.1 µM in three different cell lines (*p* < 0.05). Interestingly, the strongest response for STAT3 inhibition was observed for BxPC-3, a cell line with high IL-4 expression (Figure 5A). Additionally, PANC-1 wildtype cells were co-incubated with exogenous IL-4 (5 nM) together with *LLL12* (0.3 µM). Here, STAT3 inhibition (0.3 µM) also prevented IL-4 (5 nM) induced growth promotion (Figure 5B).

### 2.6. Suppression of IL-4Rα Inhibits Tumor Growth In Vivo

**Figure 6:**
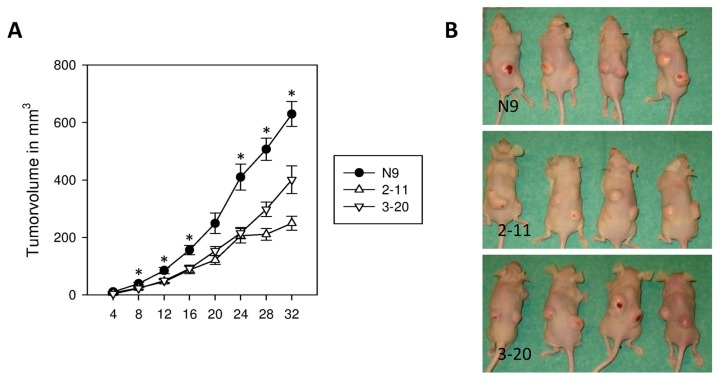
Tumor growth in a subcutaneous xenograft model. (**A**) Cells of N9, 2-11, and 3-20 were subcutaneously injected into nude mice. Tumor growth was measured every 4 days and tumor volume was calculated as V = length × height × width × ½. Mice were euthanized after 32 days. * *p* < 0.05 (N9, 2-11, 3-20; *n* = 16); (**B**) Examples of tumor formation.

In vivo tumor formation and growth of subcutaneously (s.c.) injected clones of control cells N9 and IL-4Rα knockdown cells 2-11, and 3-20 was monitored every 4 days. Tumor formation was observed at every injection site. N9 showed an accelerated growth from the beginning with significantly larger tumors compared to 2-11 and 3-20 starting 8 days after injection (*p* < 0.05). On day 32 after injection tumors of N9 reached a final volume of 630 ± 43 mm^3^ SEM. 2-11 with 250 ± 24 mm^3^ SEM and 3-20 with 401 ± 48 mm^3^ SEM only reached 39.7% respectively 63.7% of the size of control tumors (*p* < 0.001) (Figure 6A,B).

### 2.7. Morphology and Immunohistochemical Staining of Xenograft Tumors

**Figure 7:**
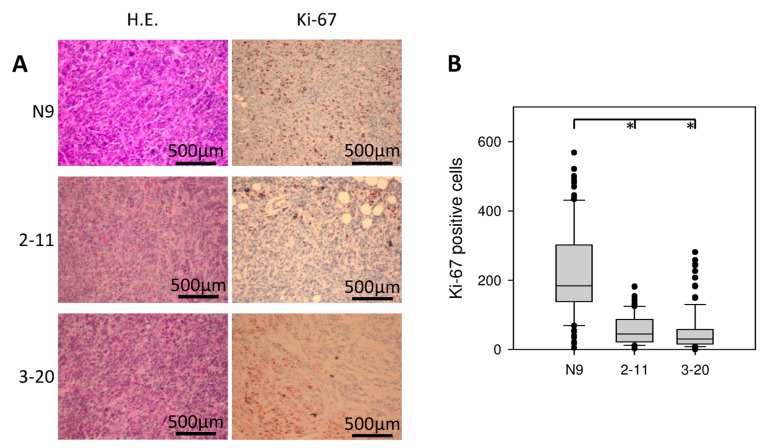
Microscopical analysis of xenograft tumors. (**A**) IL-4Rα downregulation was without effect on tumor morphology (left panels). Ki-67 immunohistochemistry was performed for all tumors (right panels); (**B**) Box plots of Ki-67 immunohistochemistry (N9: *n* = 100; 2-11: *n* = 114; 3-20: *n* = 106), revealing reduced proliferation after IL-4Rα knockdown (* *p* < 0.001).

Xenograft tumors were explanted and analyzed histologically. The morphology was not altered by IL-4Rα downregulation. All tumors showed a relatively well differentiated, papillary growth pattern with duct-like structures (Figure 7A). All tumors showed one or more central necrotic areas. Invasion of surrounding soft tissue like musculature was also observed regularly in all investigated cell lines. Reduced tumor sizes were matched to decreased tumor cell proliferation, as demonstrated by immunohistochemical staining for Ki-67. N9 showed the strongest proliferation rate with a mean amount of 223 ± 13.7 SEM Ki-67 positive cells per high power field. A significantly reduced proliferation rate was observed in 2-11 (56 ± 4.1 Ki-67-pos. cells) and 3-20 (52 ± 5.9 Ki-67 pos. cells) (*p* < 0.001) (Figure 7A,B).

## 3. Discussion

IL-4 in its physiological function is an immunomodulatory cytokine involved in T-helper (Th)-cell differentiation towards a Th-2 response [17]. Surprisingly, increased levels of IL-4 were also detected in the micromilieu of different tumors produced either by tumor cells themselves or by infiltrating lymphocytes [5,11,12,14,18]. One of its receptor chains, IL-4Rα, was shown to be overexpressed in several solid human tumors and was associated with locally advanced tumor staging, increased propensity for metastases and poor overall survival [19,20,21]. Interestingly, pro- as well as anti-oncogenic effects have been reported for IL-4 and IL-13 in malignancies of different and even of similar origin [10,12,15,16]. 

A possible explanation for the varying effects of IL-4 and its receptor chains may be their influence on multiple factors in human tumors in vivo. These include infiltrating lymphocytes and their anti-tumor response, tumor-induced angiogenesis and protection from apoptosis. Furthermore, in addition to these different targets, the caused effects in each target cell may still be dependent on the source, dose and expression time of IL-4 [22].

Focusing on pancreatic cancer, exogenous IL-4 and IL-13 increased the growth of cultured cancer cells, possibly by stimulating growth-promoting pathways such as MAP-kinases [12]. Targeting IL-4Rα with a fusion protein of IL-4 and *Pseudomonas exotoxin* resulted in strong anti-tumor activity in vitro as well as in vivo [4]. The present study aimed to address the role of IL-4Rα in growth and migrating properties of pancreatic cancer in vitro and in vivo. As it has not yet been studied previously, we used an endogenous approach by knockdown of IL-4Rα. This way, we were able to examine the oncogenic effects of the IL-4-signaling-axis without exogenous influence and thus as close as possible to its in vivo influence in growth and spread of pancreatic cancer. 

We detected both receptor chains participating in the type-II-IL-4-receptor (140 kDa IL-4Rα and 47 kDa IL13Rα1) in 7 different pancreatic cancer cell lines, derived from both pancreatic primaries (BxPC-3, MIAPaCa-2, PANC-1) as well as from metastases (AsPC-1, Capan-1, COLO-357, T3M4) [23]. The expression of the type-I-IL-4-receptor chains has been examined before by our group. Five of six tested cell lines expressed the common γ-chain, with the exception of MIAPaCa-2. While all tested cell lines also expressed IL-13Rα1, MIAPaCa-2 was the only cell line to express the decoy receptor chain IL-13Rα2 [5].

shRNA-based suppression of IL-4Rα in Capan-1 resulted in significantly reduced tumor cell growth in vitro. Interestingly, while the individual size of non-adherent colonies was markedly smaller, the formation of vital colonies was increased after IL-4Rα inhibition. The reason for this is still unclear but was well reproducible with four independent experiments demonstrating the same result. Still, we were also able to show significantly reduced tumor size in vivo. Tumor formation was not altered and all injected sites developed tumors irrespective of IL-4Rα expression. Together with the observation that IL-4Rα-downregulated tumors exhibited lower Ki67 levels, the IL-4 receptor pathway in pancreatic cancer cells seems to stimulate cell proliferation.

Our results correlate with other in vivo studies of epithelial cancer cells with IL-4-stimulated growth. In prostate cancer, blocking of IL-4-pathway reduced Ki67 expression, and in colon cancer IL-4-withdrawal strongly increased chemotherapy-induced apoptosis. Both studies were able to associate larger in vivo tumor formation with functioning IL-4 signaling [24,25]. 

Additionally, while different pancreatic cancer cell lines showed no profound differences in migration [26,27], the reduced IL-4Rα expression lead to impaired directional as well as non-directional cell movement in our experiments. Hereby these results may provide the mechanistic explanation on the molecular level of previously obtained results that demonstrated an increased risk for lymph node metastases in a human pancreatic cancer specimen with high Interleukin-4 receptor expression [28]. 

In several human tumors, IL-4 has been suggested to induce the activation of MAP- and Akt-kinases as well as STAT3, resulting in growth promotion [12,24]. These previous studies showed kinase phosphorylation in response to exogenous IL-4. They also were able to neutralize the stimulating effects of IL-4 by adding specific cytokine- and kinase-inhibitors. Furthermore, IL-4 increased the expression of anti-apoptotic proteins leading to promoted cell survival [11] and mediated the downregulation of cell adhesion molecules, promoting invasiveness [10]. In this context, the role of STAT3 needs to be stressed. Especially on non-hematopoietic cells, IL-4 will activate STAT3 through the type-II-IL-4-receptor [29]. In gastric, colorectal and pancreatic cancer STAT3 promotes tumorigenesis and is associated with shortened survival [30,31,32]. This may be due to its central role in signaling pathways. Besides being constitutively activated, multiple ligands like growth factors (EGF, TNFα) and cytokines (IL-4 and -6) converse in STAT3-activation. In turn, activated STAT3 is capable of stimulating pro-oncogenic pathways in cell survival, apoptosis, invasion, and tumor immunosurveillance [9,33].

In our experiments, IL-4 stimulation of Capan-1-wildtype cells resulted in kinase- and messenger-phosphorylation of pro-oncogenic pathways. Furthermore, the knockdown of IL-4Rα impaired IL-4 signaling. For the first time we were able to show the endogenous role of IL-4Rα in cell signaling, as previous studies used exogenous inhibitors. These results offer a possible explanation for our findings regarding reduced growth and migration abilities. IL-13 stimulation showed similar but less prominent results in wildtype cells and was not influenced by IL-4Rα-knockdown, possibly because the main target chain, IL-13Rα1, was not influenced by IL-4Rα downregulation.

To underline these results, we used the specific inhibitor of STAT3 phosphorylation, *LLL12*, in in vitro experiments [34]. Even low concentrations of up to 0.1 µM resulted in significant inhibition of cell survival in 3 different pancreatic cancer cell lines. Interestingly, the cell line with the highest IL-4Rα expression (BxPC-3) was the most susceptible to STAT3-inhibition while PANC-1 and Capan-1-clone 3-20 (both cell lines with low IL-4Rα expression) were the most resistant. Exogenous IL-4 was not able to overcome the inhibition of cell survival induced by STAT3 inhibition. These results suggest that STAT3 is a major target of IL-4 and STAT3 may be a useful additional therapeutic target, especially in cells with strong IL-4 expression.

Nevertheless, IL-4Rα-knockdown also rendered the cells more susceptible to IGF-I stimulation, indicating that impaired IL-4 signaling may be compensated by other mitogens abundantly present in the microenvironment of the tumor cells in vivo. Depending on their origin, these include growth factors like FGF (fibroblast growth factor) and VEGF (vascular endothelial growth factor), secreted by pancreatic stellate cells as well as cytokines like IL-6 originating from infiltrating immune cells [35,36]. Such interference may also play a role in not yet sufficiently explainable findings like the increase of colony formation we showed.

Besides standard chemotherapeutics, these pathways were addressed in therapeutic intentions. Interestingly, due to the mentioned interactions, a combination of different inhibitors was particularly promising. By targeting the MAPK-pathway on different levels, drug resistance was reduced [37]. Similarly, a combined inhibition of Notch and STAT3, also connected pathways, was proven more effective compared to monotherapy [38]. This again underlines the importance of messenger interactions in tumor progression as well as in the development of anticancer drugs. 

Still, the described results may be cell-type specific or varying degrees in other cell lines. Combining our new results with former results by our group [12], an association between different expressions of receptor chains and different effects of IL-4 stimulation in pancreatic cancer can be suggested. 

Further studies are warranted concerning cytokine signaling, especially concerning the role of competitive messengers. We believe this to be relevant to better understand the importance of IL-4 in the complex microenvironment of human tumors.

## 4. Materials and Methods

### 4.1. Cell Culture

Human pancreatic cancer cells AsPC-1, Capan-1 and T3M4 were cultured in RPMI (Roswell Park Memorial Institute) medium, COLO-357, MIAPaCa-2, and PANC-1 were cultured in DMEM (Dulbecco’s Modified Eagle’s Medium), and BxPC3 was cultured in RPMI and DMEM (ratio 1:1). All media were supplemented with 10% fetal calf serum (FCS), penicillin G (100 U/mL), and streptomycin (100 µg/mL), and maintained in monolayer culture at 37 °C in humidified air with 5% CO_2_. The medium for cell lines containing a neomycin resistance gene was supplemented with 1.2 mg/mL geneticin (G418).

### 4.2. Transfection

Capan-1 cells were stably transfected using 4 different plasmids each containing a shRNA construct directed against human IL-4Rα and 1 negative control plasmid (SureSilencing shRNA Plasmid for Human IL-4R by QIAGEN GmbH, (Hilden, Germany) with the Effectene Transfection Reagent Kit (QIAGEN GmbH) using the conditions described by the supplier. After transfection, cells were incubated in a medium containing G418 (1.2 mg/mL) until single colonies formed. Each clone was isolated and cultured separately until screening with immunoblot for IL-4Rα-knockdown.

### 4.3. Immunoblotting

Exponentially growing cells were washed twice with ice-cold PBS (Phosphate Buffered Saline) and lysed in the indicated cell lysis buffer (buffer composition is described in the Tables S3 and S4). To analyze N-linked protein glycosylation, Capan-1 cells were incubated in the presence or absence of tunicamycin (10 µg/mL) for 24 h before preparation of lysates. To analyze associated signaling pathways, cells were seeded at the same density. After 24 h they were serum starved with a medium containing 1.5% FCS for 24 h. Afterwards cells were stimulated with IL-4, IL-13, and IGF-I (5 nM) for 5 min or left untreated as negative control before preparing lysates. Cell lysates were subjected to SDS-PAGE (sodium dodecyl sulfate polyacrylamide gel electrophoresis) and electro-transferred to PVDF (polyvinylidene fluoride) membranes. After blocking, the membranes were blotted with the respective antibodies and the corresponding secondary horseradish-conjugated antibody. Bound antibodies were visualized using enhanced chemiluminescence. To confirm equal loading, membranes were re-blotted with an anti-β-actin antibody. Quantitative analysis was performed with ImageJ v1.47.

### 4.4. Cell Growth Assays

Anchorage-dependent cell growth was determined by the MTT colorimetric growth assay as described earlier [39]. To determine basal growth, 5000 cells/well were plated and incubated for 24 h in 200 µL complete medium before MTT solution (0.625 mg/mL) was added. Basal anchorage-independent growth was assessed by a double-layer soft agar assay. Briefly, 10,000 cells were suspended in a complete medium containing 0.35% agar and seeded in triplicate in 6-well plates onto a base layer of complete medium containing 0.9% agar. After 21 days, 6 pictures/well were taken at a 4× magnification and colony size was measured using ImageJ 1.47v. Afterwards, MTT solution (5 mg/well) was added and the 6-well plates were scanned. Viable colonies were counted in 4 cm^2^ by using ImageJ v1.47.

To analyze the effects of STAT3 inhibition, 5000 cells per well were seeded for Capan-1, PANC-1, and BxPC-3 wild-type cells as well as IL-4Rα1 knockdown cells 3-20. After 24 h, the medium was removed and the inhibitor of STAT3 phosphorylation *LLL12* was added in rising concentrations of 0.01, 0.1, 0.3, 0.5 and 1 µM. DMSO was used as control. In order to test possible antagonizing effects of IL-4, 0.3 µM of *LLL12* were combined with 5 nM of IL-4. After 48 h of incubation, MTT solution was added as described.

### 4.5. Cell Cycle Analysis

Cells of Capan-1 wildtype, negative control clones, and IL-4Rα knockdown clones were seeded at the same density. Exponentially growing cells at 60–80% confluency were taken for analysis as described before [12].

### 4.6. Cell Migration Assay

The modified Boyden chamber assay was performed as described previously [40]. Polycarbonate membranes with 8 µm pore size were used according to the protocol of the manufacturer (Corning Inc., Corning, NY, USA). The bottom chamber included a 0.6 mL medium containing 10% FCS. Cells (5 × 10^4^ suspended in 0.1 mL of medium containing 1% FCS) were seeded into the upper chamber and migration was allowed for 36 h at 37 °C. After staining, fluorescence micrographs were taken with the Olympus IX81 microscope at 10× magnification. 6 pictures per membrane were taken and migratory cells were counted using ImageJ 1.47v.

### 4.7. Single-Cell Movement Assay

Cells (40,000 per well) were seeded into a 12-well plate. After attachment, cell movement was monitored for 24 h by an Olympus IX81 motorized inverted microscope taking pictures every 20 min. The total distance of individual cells covered within 24 h was determined using ImageJ 1.47v and at least 40 cells per cell line were tracked in each experiment.

### 4.8. In Vivo Tumorigenicity Assay

All mice were housed and handled in accordance with official regulations for the care and use of laboratory animals (UKCCCR Guidelines for the Welfare of Animals in Experimental Neoplasia). Approval of all mouse experiments was granted by the Regierungspräsidium Tübingen (permission number 1203, 21 October 2014). Mice were kept under barrier conditions with a 12 h light/dark cycle and access to food and water ad libitum. To determine the effect of reduced IL-4Rα protein expression on tumorigenicity, 2 × 10^6^ of indicated cells (transfected clones of Capan-1 N9, 2-11 and 3-20) were injected subcutaneously (s.c.) into the flanks of 4–6-week old, female, athymic (nude) mice (Charles River Laboratories International, Inc., Wilmington, MA, USA). The animals (*n* = 8/group) were monitored for tumor formation every 4 days. Tumor size was measured in three dimensions and tumor volume was determined by the equation

Vol = length × width × height × ½

Mice were sacrificed 32 days after injection, or in the case of tumor size (>15 mm largest diameter) or occurring skin ulceration earlier than day 32.

### 4.9. Microscopic Analysis of Xenograft Tumors

Subcutaneous tumors were removed, formalin-fixed and paraffin-embedded. 1 µm sections were prepared from the largest diameters of each tumor followed by H&E staining or immunohistochemical analysis. Immunohistochemistry was performed as described previously [41]. Monoclonal anti-Ki-67 (Abcam ab92742, 1:500, Cambridge, UK) was used as first antibody, followed by incubation with anti-rabbit immunoglobulins conjugated to peroxidase-labeled dextran polymers (*N*-histofine H; Nichirei Corporation, Tokyo, Japan). Staining was performed with 3-amino-9-ethylcarbazol (AEC; DAKO, Glostrup, Denmark) and counterstained with haematoxylin. Analysis of Ki-67 staining was carried out in a representative peripheral area of each tumor without necrosis using high-power field microscopy.

### 4.10. Statistics

Statistical analysis and diagram creation were performed using SigmaPlot 12.0 (Systat Software, Inc., Chicago, IL, USA). Results are expressed as mean ± SEM and the Student’s *t-*test and the Mann-Whitney Rank Sum test were used for statistical analysis (two-sided). *p* < 0.05 was taken as the level of significance. For the in vivo assay, we hypothesized a reduction of tumor volume of at least 30% in order to reach clinical significance. To detect this difference (α = 0.05, power = 80%) a total of 16 tumors per cell clone (8 animals/clone) were required.

## 5. Conclusions

In summary, our results clearly demonstrate that signaling via IL-4Rα can induce mitogenic signaling and promote the malignant phenotype of human pancreatic cancer cells. For the first time we were able to show that these effects are present even without exogenous stimulation, possibly due to auto- and paracrine stimulation or IL-4Rα being constitutively activated. We also have shown the examined effects being caused by activation of known growth- and invasion-promoting signaling pathways. Future projects may aim to determine further aspects of this pathway regarding IL-4 induced chemotherapy resistance and interactions with neighboring cells, especially focusing on pancreatic cancer stem cells. Pharmaceutical modification of these pathways may eventually contribute to the treatment of this dismal disease.

## Supplementary Materials

Supplementary materials can be found at www.mdpi.com/1422-0067/18/4/716/s1.

## Abbreviations

IL-4/-13	Interleukin-4/-13
IL-4Rα	Interleukin-4-receptor-α-chain
IL-13Rα1	Interleukin-13-receptor-α-1-chain
*LLL12*	Specific inhibitor of STAT3 phosphorylation
N9, N10	Capan-1 cells carrying a negative control plasmid
2-11, 3-20	Capan-1 cells transfected with shRNA targeting IL-4Rα
