# Supplementary material for: Endogenously Expressed IL-4Rα Promotes the Malignant Phenotype of Human Pancreatic Cancer In Vitro and In Vivo
Source: Int J Mol Sci. 2017 Mar 28;18(4):716. doi: 10.3390/ijms18040716 (PMC5412302; doi:10.3390/ijms18040716)
Supplement: Supplementary file 1 [file ijms-18-00716-s001.pdf]

Supplementary Materials: Endogenously Expressed IL-4Rα Promotes the Malignant Phenotype of Human Pancreatic Cancer In Vitro and In Vivo

Benno Traub, Lie Sun, Yongsu Ma, Pengfei Xu, Johannes Lemke, Stephan Paschke, Doris Henne-Bruns, Uwe Knippschild, and Marko Kornmann

1. IL-4Rα glycosylation, RT-PCR control

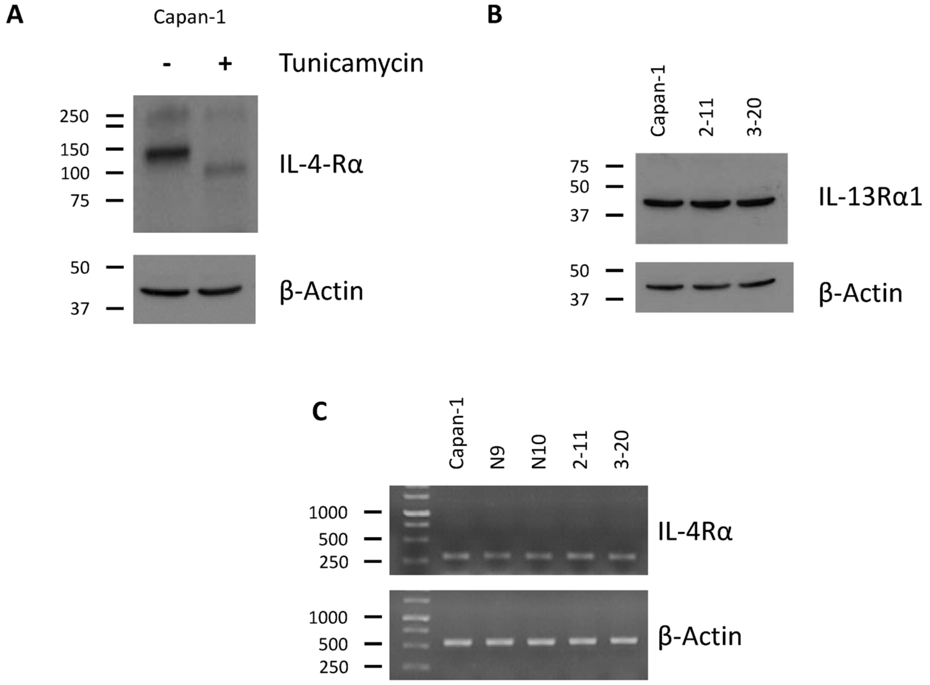

Figure S1. Receptor glycosylation of IL-4Rα changes band movement from approx. 90kDa to 140kDa (glycosylated receptor) as shown with Tunicamycin (A). Transfection of Capan-1 with an IL-4Rα-shRNA construct was without effect on IL-13Rα1 expression in knockdown clones (B) and was without effect on RNA expression (C).

2. Cell cycle analysis

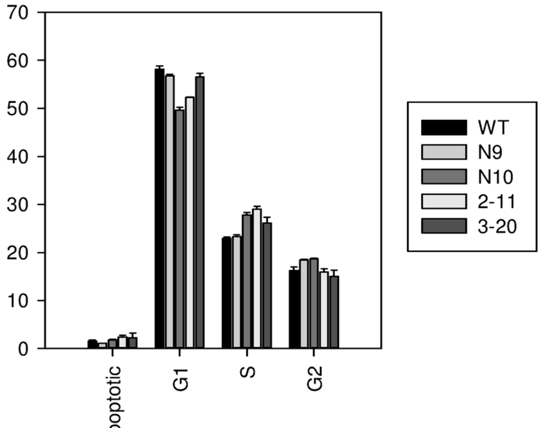

Figure S2. Cell cycle analysis shows no difference in cell cycle progression after IL-4 knockdown.

3. Capan-1 cytoskeleton structure

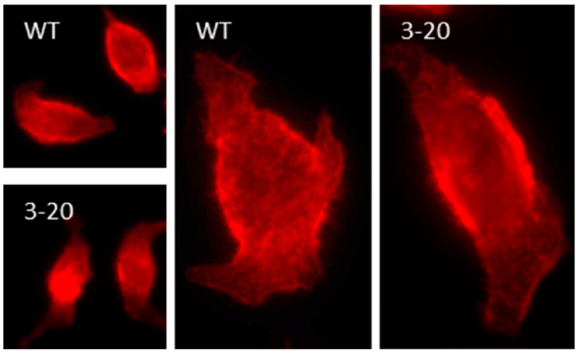

Figure S3. Phalloidin staining of actin fibers showed no differences in actin arrangement after IL-4Rαknockdown (40× (low) and 96× (high) magnification).

4. Effect of IL-4Rα downregulation on IL-4 and IL-13 signaling – Quantitative analysis

After developing the western-blot membranes, the bands were scanned and the density was analyzed by using ImageJ v1.47.

In WT-cells, phosphorylation after cytokine-stimulation was compared to untreated WT-control-cells. In order to analyze the relative intensity of phosphorylation after downregulation of IL-4Rα, for each messenger the signal of N10 was defined as 100% and the corresponding band of 3-20 was set in proportion

The following Figure S4 demonstrates the calculation of the quantitative analysis which will be shown in Figure S5 and Table S1 (Phosphorylation in WT cells) as well as Figure S6 and Table S2 (Relative Phosphorylation in 3-20 compared to N10)

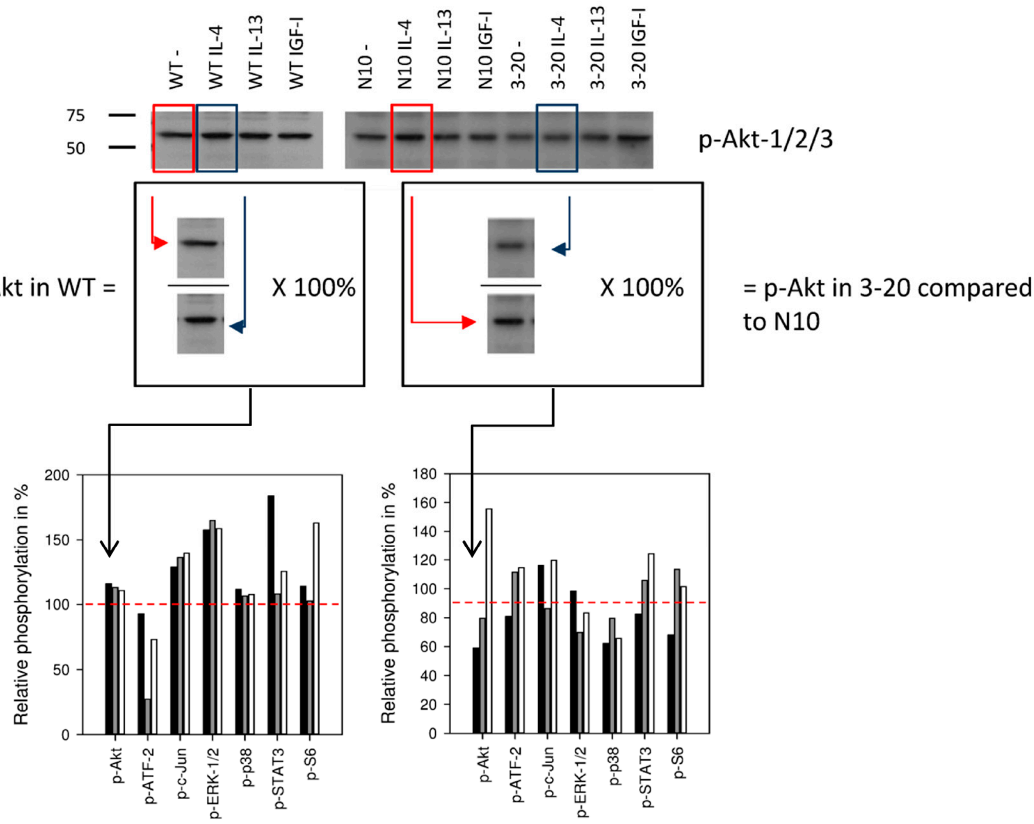

Figure S4. Calculation of the quantitative analysis

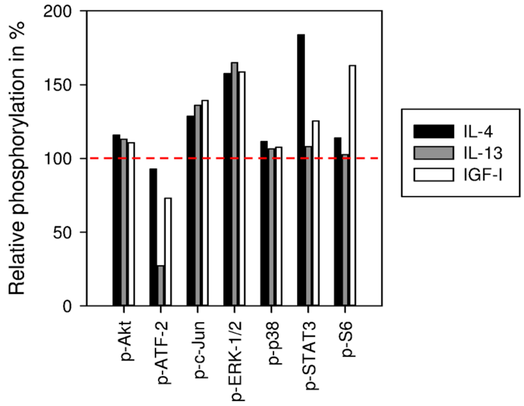

Figure S5. Relative phosphorylation of Capan-1-WT cells after cytokine stimulation

WT cell phosphorylation bands were scanned and the density was compared to respective untreated control for each cytokine. The red dashed line represents the signaling intensity of untreated WT cells

Table S1. Relative phosphorylation of Capan-1-WT cells after cytokine stimulation.

Cytokine	p-Akt-1/2/3		p-ATF-2		p-c-Jun	p-ERK-1/2	p-p38	p-STAT3	p-S6
IL-4	116%		93%		129%	158%	112%	184%	114%
IL-13	113%		27%		136%	165%	107%	108%	103%
IGF-I	111%	73%	139%	159%	108%	125%		163%	

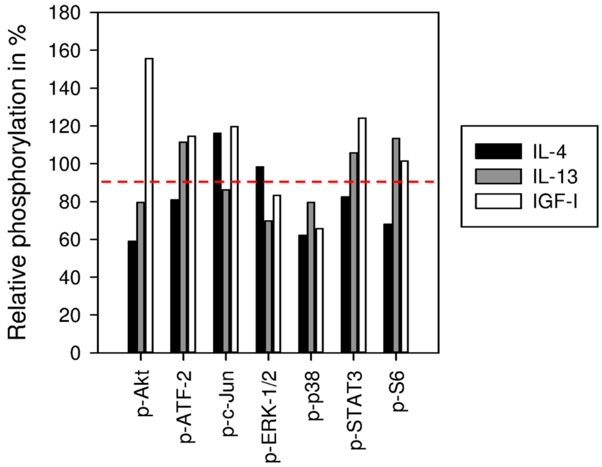

Figure S6. Relative phosphorylation of 3-20 compared to N10.

Here, the relative phosphorylation after downregulation of IL-4Rα is shown. For each messenger, signal of N10 was defined as 100% (red dashed line) and the corresponding band of 3-20 was set in proportion.

Table S2. Relative phosphorylation of 3-20 compared to N10.

Cytokine	p-Akt-1/2/3	p-ATF-2	p-c-Jun	p-ERK-1/2	p-p38	p-STAT3	p-S6T
IL-4	59%	81%	116%	98%	62%	82%	68%
IL-13	79%	111%	86%	70%	80%	106%	113%
IGF-I	156%	115%	120%	83%	66%	124%	101%

5. Composition of lysis-buffers used in immunoblotting:

Table S3. SDS-Lysisbuffer (Used for proteins, targeted by antibodies without phosphorylation-dependent epitope).

Tris-HCl 1,25M pH 6.6	100µl
Sodium dodecyl sulfate (SDS) 10%	90µl
Aprotinin 0.25mg/ml	10µl
Pepstatin A 1mg/ml	10µl
Benzamidine 100mM	10µl
Phenylmethanesulfonyl fluoride (PMSF) 200mM	5µl
Aqua dest.	775µl

Table S4. Lysisbuffer, used for extraction of phosphorylated proteins

Lysisbuffer (Stock solution) (Stock: 1.5ml Tris (1M, pH 7.4), 1.5ml NaCl (5M), 0.5ml Triton 100%, 5ml Glycerol 99%, 41.5ml H20 + 1 tablet cOmplete (Roche GmbH))	1ml
Dithiothreitol (DTT) 1M	1µl
PMSF 100mM	5µl
Sodium orthovanadate (SOV) 0.1M	10µl
β-Glycerol-phosphate (β-GP) 0.1M	10µl
Sodium fluoride (SF) 0.5M	100µl
Aqua dest.	775µl
